# Yawning as a Rare Side Effect With Increased Escitalopram Dose: A Case Report

**DOI:** 10.7759/cureus.61559

**Published:** 2024-06-03

**Authors:** Nayan Sinha, Ragini Patil, Rishitha Kotla, Namita Sahu

**Affiliations:** 1 Department of Psychiatry, Jawaharlal Nehru Medical College, Datta Meghe Institute of Higher Education and Research, Wardha, IND

**Keywords:** selective serotonin reuptake inhibitor (ssri), antidepressants and yawning, drug-induced yawning, escitalopram, yawning

## Abstract

Yawning is a normal physiological process that occurs naturally in all human beings in different settings, such as hunger, drowsiness, or stress. It is typically harmless, but abnormal yawning can be seen in many medical conditions. In psychiatry, it frequently occurs in disorders like depression, insomnia, and anxiety due to disturbed sleep. It has also been observed as an adverse reaction of some drugs, like escitalopram, a selective serotonin reuptake inhibitor. Escitalopram is a widely prescribed, well-tolerated antidepressant and antianxiety drug that can induce a range of side effects, one of which is excessive yawning. Its excessive occurrence can be distressing for patients, affecting their socio-occupational functioning. Clinically, differentiating yawning induced by escitalopram treatment from that in depression can be a diagnostic hurdle. Awareness and recognition of this lesser known side effect can improve patient outcomes by allowing for timely adjustments and easing the discomfort.

## Introduction

Yawning is a normal physiological function that is observed from early human development through adulthood. It becomes less frequent as individuals mature. There are a variety of contexts where yawning is commonly present, such as before or after eating, after alcohol ingestion, in motion sickness, or changes in altitude. Other situations include monotony, hunger, and tiredness [1]. Although numerous factors like nitric oxide, glutamate, gamma-aminobutyric acid, serotonin, adrenocorticotropic hormone, melanocyte-stimulating hormone, and sexual hormones influence yawning, dopamine plays a significant role. As dopamine is released, it triggers the production of oxytocin in the hypothalamus's paraventricular nucleus. As a result, oxytocin stimulates cholinergic transmission in the hippocampus, which leads to the activation of muscarinic receptors via acetylcholine, ultimately leading to yawning [2].

Pathological yawning can occur in various medical conditions, including neurological, psychiatric, infectious, and iatrogenic [1]. Concerning psychiatry, yawning often occurs when feeling tired and becomes more frequent when external stimuli fail to provide alertness. This phenomenon has been linked to depression due to symptoms like disrupted sleep patterns and fatigue [3]. However, few studies have even identified yawning as a rare and unexpected side effect of certain antidepressant medications [3-6]. There have been articles linking it specifically with antidepressants, including fluoxetine, paroxetine, sertraline, escitalopram, duloxetine, venlafaxine, imipramine, and clomipramine [7-9]. This could be secondary to the sedative effects of the drug or could be an independent adverse effect. It typically manifests a few days to weeks following the initiation of treatment and tends to diminish upon dosage reduction or cessation of the medication [7].

Escitalopram is commonly prescribed for the treatment of various psychiatric disorders, like depression, anxiety, panic, and obsessive-compulsive disorder. It is usually well tolerated, but a range of side effects, including sexual dysfunction, gastrointestinal disturbances, and sleep disturbances, are associated with this drug [10]. Yawning is a lesser known side effect of escitalopram. Few articles document the occurrence of yawning as an adverse effect resulting from drug administration. This article presents a case of escitalopram-induced yawning and discusses the diagnostic dilemma and management strategies.

## Case presentation

A 28-year-old married female, a nurse by profession from a low socioeconomic joint family, came with complaints of pervasive low mood, loss of interest in pleasurable activities, and feeling fatigued throughout the day. These symptoms had been present for the past four months due to marital problems involving frequent altercations with her husband over financial issues. Additionally, she experienced decreased self-esteem and negative thoughts about the future. Her appetite had decreased over the past four months, and she had difficulty initiating and maintaining sleep, leading to socio-occupational impairment. She had a well-adjusted premorbid personality, with no medical comorbidities and no history of psychiatric or neurological illness in the family.

The patient was diagnosed with a moderate single-episode depressive disorder without psychotic symptoms, according to the International Classification of Diseases-11 [11]. Her routine investigations, including complete blood count, liver function test, kidney function test, thyroid profile, fasting blood sugar, serum vitamin B12, serum vitamin D, electrocardiogram, urine routine, and culture, were within normal limits. She was started on a 5 mg dose of tablet escitalopram once nightly, which was gradually increased to 15 mg on day 20. The tablet clonazepam 0.5 mg was started once nightly, and it was tapered and discontinued once her sleep improved on day 7. The patient showed improvement in her symptoms.

However, it was observed that as the dose of escitalopram increased to 15 mg, she began to yawn more frequently, approximately five to six times every 15 minutes. By then, clonazepam had been tapered and stopped (13 days before increasing the dose of escitalopram to 15 mg). Initially, it was thought that the yawning was due to fatigue or sedation, but she experienced no sleep disturbances. In fact, her sleep pattern had improved, and she no longer felt fatigued. Despite this, she had an irresistible urge to yawn, which irritated her and interfered with her daily activities and work life. When the dose of escitalopram was further increased to 20 mg once nightly, the yawning spells increased to seven to eight times every 15 minutes. The patient had no medical comorbidities and was not taking any other medications. Due to the temporal correlation, it was suspected that escitalopram was causing the excessive yawning. The yawning spells decreased and eventually disappeared when the dosage was reduced gradually. Later, the patient was managed with tablet escitalopram 10 mg, once nightly. Along with it, supportive psychotherapy and couples counseling sessions were taken. She was followed up weekly. No adverse effects were reported, and the patient reported 100% improvement.

## Discussion

Yawning, a frequently occurring physiological occurrence, is typically linked with boredom or insomnia and has never been widely recognized or acknowledged as a side effect of any medication. Consequently, it often receives little attention, leading to frequent underreporting. Pathological yawning (greater than three per 15 minutes) can be observed in various conditions, as summarized in Table 1 [1,12].

**Table 1:** Pathological etiologies of yawning

Serial number	Etiology	Examples
1	Psychiatric disorders	Depression, insomnia, and anxiety
2	Neurological conditions	Parkinson's, stroke, narcolepsy, and epilepsy
3	Gastrointestinal issues	Gastroduodenal ulcer, gastroesophageal reflux, and pharyngeal obstruction
4	Metabolic disorders	Acidocetosis, renal insufficiency, severe hepatic failure, and thyroid insufficiency
5	Infectious diseases	Encephalitis and meningitis
6	Latrogenic factors	Opiate withdrawal, antidepressant use, and sodium valproate administration

The medications that can trigger yawning include selective serotonin reuptake inhibitors, serotonin and norepinephrine reuptake inhibitors, tricyclic antidepressants, dopaminergic drugs, monoamine oxidase inhibitors, and benzodiazepines, as described in Table 2 [13].

**Table 2:** Psychotropics associated with yawning

Serial number	Psychotropics	Examples
1	Selective serotonin reuptake inhibitors	Fluoxetine, paroxetine, and escitalopram
2	Serotonin and norepinephrine reuptake inhibitors	Venlafaxine and duloxetine
3	Tricyclic antidepressants	Imipramine and clomipramine
4	Dopaminergic drugs	Levodopa, pramipexole, and apomorphine
5	Monoamine oxidase inhibitors	Selegiline, isocarboxazid, and phenelzine
6	Benzodiazepines	Clonazepam

In this patient's case, pathological yawning occurred following an increase in the dosage of escitalopram tablets. Her frequency of yawning was observed to be directly proportional to the dose. She was not taking any other medications and did not experience fatigue or drowsiness. No other underlying causes for her yawning were identified. Her symptoms decreased when the dosage was lowered, and they eventually ceased altogether. To eliminate the possibility of a psychogenic cause, the patient was given a placebo after lowering the dose of the drug, but her symptoms did not reappear. The Naranjo adverse drug reaction probability scale was utilized to assess the patient, revealing a score of 8, suggesting escitalopram as the most probable cause of the patient's yawning [14].

While uncommon, few articles do report the induction of yawning with initiation or increase in the dose of escitalopram treatment [15]. The exact mechanism remains unclear, but researchers have proposed several hypotheses based on the drug's effect on neurotransmitter systems in the brain. One theory suggests that elevated serotonin levels in the raphe nucleus may reduce dopamine activity within the basal ganglia. Another hypothesis proposes a temporary disruption in frontal lobe function due to changes in serotonin levels. Since the frontal lobes play a role in various functions like arousal and alertness, any potential impairment could manifest in yawning [16]. The patient was continued with the same antidepressant at a lower dose, which did not induce any side effects and proved to be efficacious when combined with supportive psychotherapy and couples counseling.

## Conclusions

For those being treated with antidepressant medication, identifying whether yawning is caused by the medication or is a symptom of depression presents a diagnostic hurdle. Yawning induced by antidepressants typically coincides temporally with the initiation of treatment and the onset of side effects. Clinicians must carefully evaluate the timing and recurrence pattern of yawning episodes alongside other related symptoms. While escitalopram is widely considered safe and efficacious in managing various psychiatric conditions like depression and anxiety, clinicians should remain aware of the possibility of this uncommon side effect.
